# Tau and Aβ imaging, CSF measures, and cognition in Alzheimer's disease

**DOI:** 10.1126/scitranslmed.aaf2362

**Published:** 2016-05-11

**Authors:** Matthew R. Brier, Brian Gordon, Karl Friedrichsen, John McCarthy, Ari Stern, Jon Christensen, Christopher Owen, Patricia Aldea, Yi Su, Jason Hassenstab, Nigel J. Cairns, David M. Holtzman, Anne M. Fagan, John C. Morris, Tammie L. S. Benzinger, Beau M. Ances

**Affiliations:** 1 Department of Neurology, Washington University in St. Louis, St. Louis, MO 63110, USA; 2 Department of Radiology, Washington University in St. Louis, St. Louis, MO 63110, USA; 3 Knight Alzheimer's Disease Research Center, Washington University in St. Louis, St. Louis, MO 63110, USA; 4 Department of Mathematics, Washington University in St. Louis, St. Louis, MO 63110, USA; 5 Department of Pathology, Washington University in St. Louis, St. Louis, MO 63110, USA; 6 Hope Center for Neurological Disorders, Washington University in St. Louis, St. Louis, MO 63110, USA; 7 Department of Neurosurgery, Washington University in St. Louis, St. Louis, MO 63110, USA

## Abstract

Alzheimer's disease (AD) is characterized by two molecular pathologies: cerebral β-amyloidosis in the form of β-amyloid (Aβ) plaques and tauopathy in the form of neurofibrillary tangles, neuritic plaques, and neuropil threads. Until recently, only Aβ could be studied in humans using positron emission tomography (PET) imaging owing to a lack of tau PET imaging agents. Clinical pathological studies have linked tau pathology closely to the onset and progression of cognitive symptoms in patients with AD. We report PET imaging of tau and Aβ in a cohort of cognitively normal older adults and those with mild AD. Multivariate analyses identified unique disease-related stereotypical spatial patterns (topographies) for deposition of tau and Aβ. These PET imaging tau and Aβ topographies were spatially distinct but correlated with disease progression. Cerebrospinal fluid measures of tau, often used to stage preclinical AD, correlated with tau deposition in the temporal lobe. Tau deposition in the temporal lobe more closely tracked dementia status and was a better predictor of cognitive performance than Ab deposition in any region of the brain. These data support models of AD where tau pathology closely tracks changes in brain function that are responsible for the onset of early symptoms in AD.

## Introduction

Alzheimer's disease (AD) is characterized neuropathologically by the presence of β-amyloid (Aβ) plaques and tau immunoreactive neuritic plaques, neurofibrillary tangles, and neuropil threads in the cerebral cortex (1). Autopsy studies demonstrate that Aβ and tau pathology accumulate in stereotypical spatial patterns, or topographies, over the course of the disease (2, 3). Topographic studies of AD-related pathology in vivo, until recently, have been limited to fibrillar Aβ (4, 5) owing to a lack of positron emission tomography (PET) imaging agents for tau pathology. However, a number of tau pathology imaging agents have recently been developed and are available for human studies (6).

Extant models of AD pathophysiology, such as the amyloid cascade hypothesis, establish a temporal ordering of markers, with the emergence of AD-related tauopathy occurring downstream to the accrual of Aβ pathology (7–11). Consistent with this proposal, markers of tau pathology correlate more closely with changes in cognition compared to Aβ measures (12). However, the prior lack of imaging agents has limited studies of tau to postmortem examinations (13–16) or measures that lack topographic information such as measurement of tau in cerebrospinal fluid (CSF). As a result, relationships between cognition and Aβ topography have been defined (17), but the relationship with tau topography is not defined in vivo. Without this spatial information, CSF tau measures may be assessing other forms of neurodegeneration related to cell death or synaptic dysfunction.

The use of both tau and Aβ imaging allows for the attribution of AD-related cognitive dysfunction to specific pathological processes and locations within the brain. Further, the spatial relationships between tau pathology and other markers of AD have not been established. Here, we examine how tau imaging topographies relate to clinical status, Aβ imaging, CSF measures of AD pathology, and neuropsychological performance. Tau and Aβ represent distinct pathological processes, but they are strongly related in the context of AD. Given that PET- and CSF-based techniques may measure similar processes (18), PET and CSF measurements of tau and Aβ may be related. Here, we assessed the relationship between tau PET imaging, Aβ PET imaging, CSF measures, and cognition in healthy control individuals and those with mild AD. Given extant models (8, 9) and limited autopsy-based empirical data (13–15), we hypothesized that tau would be a stronger predictor of cognition than Aβ owing to its position later in the preclinical disease course. These relationships were investigated in vivo using multivariate statistical models well suited to identify PET topographies associated with the presence or absence of disease. We identified the relationships between Aβ and tau pathology in distinct topographies and correlated them with measurements of Aβ and tau in CSF and neuropsychological measures of disease.

## Results

Data from 46 individuals were analyzed (36 cognitively normal controls, 10 with mild AD). Demographics of each subset of the cohort are provided in Table 1. Global cognitive and functional performance was assessed using the clinical dementia rating (CDR) scale (19). Each participant underwent T807 (tau) (20) and florbetapir (Aβ) PET imaging and magnetic resonance imaging (MRI). A subset of individuals also underwent lumbar puncture for CSF assays and neuropsychological testing. Standardized uptake value ratios (SUVRs) with (when presenting regional data) and without (when presenting voxelwise data) partial volume correction were calculated within 42 bilateral anatomical brain regions of interest (ROIs) defined by FreeSurfer software (21, 22).

### Cognitively impaired participants demonstrate elevated tau burden

**Fig. 1:**
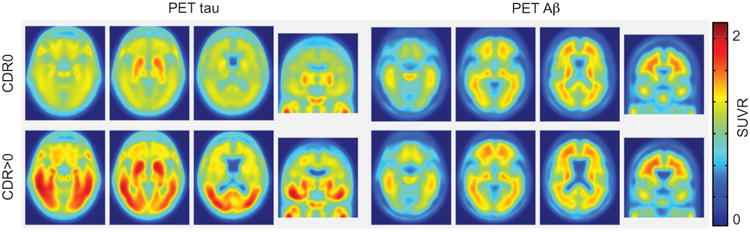
Mean tau and Aβ topographies Mean PET tau and Aβ topographies in participants with and without clinical AD. Mean tau SUVR images averaged across participants with CDR0 (top row) or CDR>0 (bottom row). Images are presented in radiological convention. These images show relatively low pathology in cognitively normal individuals and increased pathology in the cognitively impaired group.

Mean (across participants) tau and Aβ SUVR imaging data are presented in Fig. 1 (representative single subject data are shown in fig. S1). The data were split into two groups based on the presence or absence of cognitive impairment. Cognitively normal (CDR0; *n* = 36) participants showed minimal tau tracer uptake throughout the brain with the exception of the basal ganglia. Cognitively impaired (CDR>0; *n* = 10) participants exhibited markedly increased tau tracer uptake in the temporal lobes and throughout the cerebral cortex. Aβ imaging showed the known difference in measured Aβ deposition between cognitively normal and cognitively impaired groups (Fig. 1).

### Distinct tau and Aβ topographies are associated with cognitive impairment

**Fig. 2:**
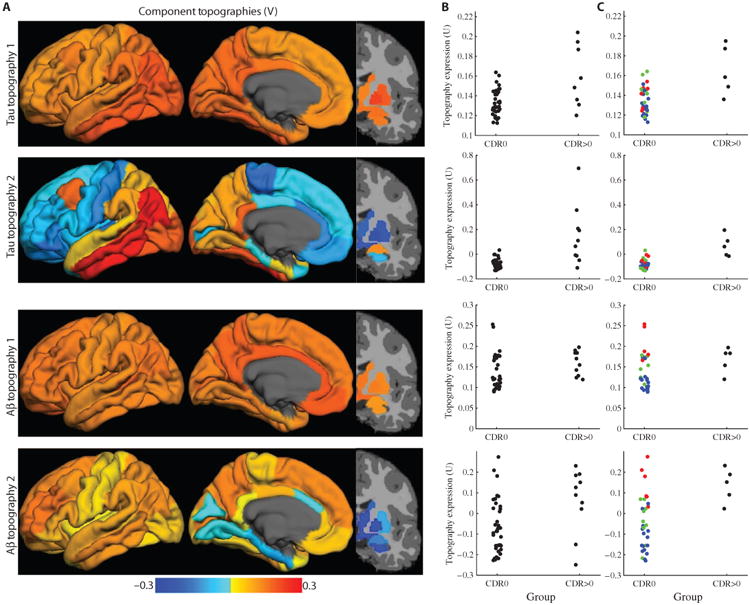
PET tau and Aβ topographies are associated with disease severity (**A**) Right singular vectors (V) of the SVD of the regional PET tau and Aβ data represented as topographies. The dimensionality of both tau and Aβ was estimated to be two. The first topography for both PET species broadly represents the mean (that is, all regions are homogeneously weighted). The second tau topography is present within the temporal lobe, and the second Aβ topography is largely seen in frontal and parietal lobes. Color bar represents regional weights within each singular component. (**B**) The representations of these topographies in individuals (left singular vectors, U) (that is, the original data projected onto these topographies) varied with CDR for both PET tau topographies and the second PET Aβ topography. Increasing disease severity (measured using the CDR) is associated with increasing representation of the present topographies in individuals. However, particularly in the Aβ data, there was significant heterogeneity. (**C**) Similar graphs as (B), but only participants with CSF analysis were included. Color in the CDR0 group indicates preclinical disease status: blue corresponds to healthy aging (that is, no Aβ or tau pathology), green corresponds to stage 1, and red corresponds to stage 2 of the CDR.

To assess the behavior of PET tau and Aβ pathology across participants, the imaging data were first decomposed into component topographies. This accomplished two goals: (i) it accounted for the correlation structure of the data across ROIs and (ii) reduced the number of statistical comparisons. Singular value decomposition (SVD) identified a small number of latent component topographies whose representation in single subjects explained a majority of the variance present in the data. In each participant, tau and Aβ pathology burden measured by PET imaging was assessed in 42 ROIs; many ROIs were highly correlated with other ROIs and could be more concisely summarized as topographies or combinations of ROIs. The PET imaging tau and Aβ data were arranged into participants (*n* = 46) by ROI matrices (*M* = 42) separately and subjected to SVD. PET imaging of tau and Aβ was best described as a combination of two component topographies (Fig. 2A). Both PET tau and Aβ imaging data were empirically determined to have two significant components (that is, rank = 2), suggesting that the two markers have similar signal-to-noise characteristics. For both PET tau and Aβ, the first topography roughly corresponded to the mean of the image (that is, regions were similarly positively weighted). The second PET tau topography was most strongly localized (or “loaded,” in SVD terminology) in the temporal lobe including the hippocampus. In contrast, the second PET Aβ topography was most strongly localized in frontal and parietal regions. This analysis demonstrated that PET imaging data for tau and Aβ exhibited strong autocorrelation across ROIs but that each had distinct topographies.

In each participant, the representation of each of the two PET tau and two PET Aβ topographies described above was best estimated using regional weights derived from SVD. For each topography, these values were subjected to an analysis of covariance (ANCOVA) with age, CDR, and presence of an APOE ε4 allele as factors (table S1). The two PET tau topographies were more strongly represented in participants with a higher CDR status (*P* < 0.001) but were not significantly modified by age or APOE status (Fig. 2B). The first PET Aβ topography was not significantly associated with any variables of interest; the second topography was more strongly represented in participants with higher CDR status (*P* = 0.04) and moderately associated with increasing age (*P* = 0.08). In both the tau and Aβ data, there were statistically significant differences between the CDR0 and CDR>0 groups; however, there was a significant overlap particularly in the Aβ data (Fig. 2C). The separation between the CDR0 and CDR>0 groups was more marked in the PET tau data compared to the PET Aβ data. This visual observation was quantified as *F* values (table S1); these *F* values were an order of magnitude larger for PET tau than for PET Aβ (table S1). Thus, as measured by the CDR, representation of tau topographies was more strongly associated with dementia status across participants.

### Evidence of preclinical AD is associated with pathological topographies

Inspection of Fig. 2B revealed that expression of each topography varied widely in the CDR0 participants, particularly with respect to PET Aβ topographies. CDR0 implies cognitive normality but not absence of preclinical pathology. Preclinical AD can be operationalized using CSF measures of tau and Aβ with simple scalar cutoffs. To most simply characterize the pathological status of the CDR0 participants, median splits were used to dichotomize participants as either CSF Aβ-positive or CSF Aβ-negative or tau-positive or tau-negative. CSF that was Aβ- and tau-negative corresponded to healthy aging without pathology. Aβ-positive tau-negative CSF corresponded to stage 1 preclinical disease; Aβ-positive tau-positive CSF corresponded to stage 2 preclinical disease (23, 24). Stratifying CDR0 participants in this manner revealed that CDR0 participants with higher expression of the component topographies tended to have evidence of elevated Aβ or tau pathology in CSF (Fig. 2C). The effect of preclinical disease stage was more marked for PET Aβ, suggesting that tau pathology burden was more closely temporally linked to clinical status as measured by CDR and preclinical disease stage. The preceding results were reproduced in a larger sample of individuals who only had PET tau data available (see Supplementary Results and tables S4 and S5).

### PET tau and Aβ, in distinct topographies, are strongly correlated

**Fig. 3:**
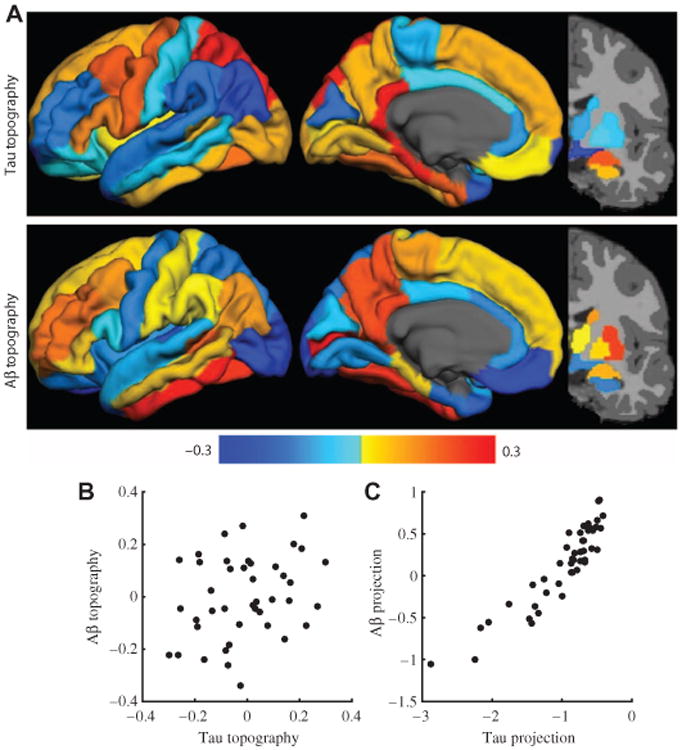
Tau and Aβ in distinct topographies are strongly correlated (**A**) Topographies for PET tau and PET Aβ imaging derived from canonical correlations that maximize the correlation between PET tau and Aβ deposition. (**B**) Scatter plot of the weights on each region demonstrates that PET tau and Aβ topographies are not significantly related. This is consistent with the typical topographies of tau and Aβ pathology being distinct. (**C**) The data projected onto the canonical variables revealed a robust positive relationship.

The previous analysis described the relationship between PET tau and PET Aβ topographies and disease stage. Next, the relationship between the PET tau and Aβ topographies was compared directly using canonical correlation. Canonical correlation is a multivariate generalization of the more familiar bivariate correlation. Canonical correlation identified both a PET tau and PET Aβ topography such that the data, when projected onto these weighted topographies, were maximally correlated. Thus, canonical correlation identified the topography of each pathological species that was mostly related to the other species. The present PET tau and PET Aβ topographies were represented by a single canonical correlation that explained 75% of the covariance in the data (Fig. 3). The weighted regional patterns of the two topographies were modestly correlated (*r* = 0.29, *P* = 0.062), which reflects the known divergence in the pathological topographies (2, 3). The PET data weighted and averaged according to these topographies were highly correlated (*r* = 0.92, *P* < 10^−18^), which reflects the strong relationship between tau and Aβ when the appropriate topographies are considered. The PET tau topography was most heavily localized in the medial temporal lobe, parietal cortex, and precuneus and was most highly correlated with PET Aβ topography in the frontal and parietal regions.

### Mean PET tau and Aβ correlate with CSF tau, phosphorylated tau, and Aβ_42_

PET tau and Aβ imaging techniques and CSF-based techniques may measure correlated pathological processes (18). Therefore, the two measures may be related. To investigate this relationship, the correlations between mean (calculated across all gray matter regions) PET tau and Aβ and CSF tau, phosphorylated tau (p-tau), and Aβ_42_ were calculated (table S2). As predicted, mean PET tau positively correlated with CSF tau (*r* = 0.36, *P* = 0.029) and CSF p-tau (*r* = 0.29, *P* = 0.089) but did not significantly correlate with CSF Aβ_42_ (*r* = −0.25, *P* = 0.14); mean PET Aβ negatively correlated with CSF Aβ_42_ (*r* = −0.54, *P* = 0.006) but also positively correlated with CSF tau (*r* = 0.52, *P* = 0.0011) and p-tau (*r* = 0.49, *P* = 0.0023). The negative correlations were due to CSF Aβ_42_ being reduced, not increased, during AD pathogenesis as more Aβ becomes deposited in the brain.

### CSF tau and Aβ_42_ correlate with specific topographies of PET tau and PET Aβ

**Fig. 4:**
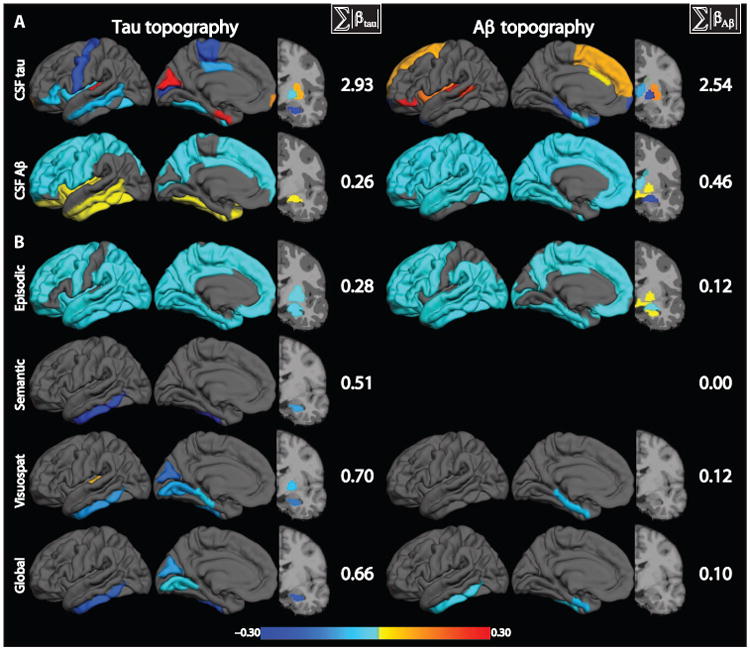
CSF and neuropsychological performance are predicted by tau and Aβ topographies Each row represents a single penalized regression model where either CSF protein or neuropsychological performance is predicted. Σ|β_tau_| and Σ|β_Ab_| represent the total predictive weight of tau and Aβ topographies, respectively. Gray regions have no predictive weight. (**A**) Penalized regression models that predict CSF tau and Aβ_42_ using tau and Aβ topographies. Because CSF Aβ_42_ is inversely related to amyloid burden, negative weights indicate regions where topographies predict worse CSF Aβ_42_ pathology. (**B**) Penalized regression models that predict neuropsychological performance in each examined domain: global, episodic, semantic, and visuospatial (visuospat). Regions with negative values (displayed in cool colors) are where more PET pathology predicts lower cognitive performance.

Penalized regression models (25–27) with either CSF tau or Aβ_42_ (separately) as an outcome and regional PET tau and Aβ SUVR (together) as predictors were fit to determine the relationship between CSF and regional PET measures. PET tau and PET Aβ topographies were considered together in the model, allowing for the data to determine the modality that best predicted the variable of interest. The critical feature of penalized regression is that the solution is sparse (that is, the regression βs corresponding to most PET tau and Aβ ROIs are set to 0), and only the most predictive ROIs receive nonzero regression β weights. The relationship between PET tau and Aβ topographies and CSF tau was examined first. The penalized model significantly fit the data (*R*^2^ = 0.96, *Z* = 4.72, *P* < 0.0001). The combination of PET tau and Aβ topographies that best correlated with CSF tau is shown in the top row of Fig. 4A (exact values are shown in table S3). CSF tau was equally related to PET tau (Σ|β_tau_| = 2.93) and PET Aβ (Σ|β_Aβ_| = 2.54), as measured by the sum of the absolute value of the regression β values corresponding to PET tau and Aβ ROIs, respectively. This equal weighting was consistent with tau pathology being dependent on initial Aβ accumulation. Predictive PET tau regions included entorhinal and temporal cortex and the cuneus. The PET Aβ contribution was derived from the temporal and frontal regions. Similar topographies of predictive PET ROIs were found when predicting CSF p-tau; the overall correlation between the CSF tau and CSF p-tau predictive topographies was high (*r* = 0.77, *P* < 10^−16^).

Next, the same relationship was investigated, but with CSF Aβ_42_ as the predicted outcome variable. The penalized model significantly fit the data (*R*^2^ = 0.34, *Z* = 3.22, *P* = 0.0006). The joint topographies of PET tau and Aβ that best correlated with CSF Aβ_42_ are shown in the bottom row of Fig. 4A (exact values are shown in table S3). Most of the regression β values were negative owing to the inverse relationship between CSF Aβ and pathology burden. In contrast to the CSF tau model, the predictive weight was more skewed toward PET Aβ (Σ|β_Aβ_| = 0.46) compared to PET tau (Σ|β_tau_| = 0.26). This suggested that PET tau has relatively less predictive information relating to CSF Aβ_42_. The contributing topography from PET tau was primarily loaded in the superior portions of the cortex. The PET Aβ topography was primarily composed of frontal and parietal regions. The highly significant predictive power of both models suggested that there was a strong relationship between CSF and PET measures of tau and Aβ pathology.

### Specific tau and Aβ topographies correlate with neuropsychological performance

To examine the relationship between pathology and cognitive function, the relationship between PET tau and Aβ topographies and neuropsychological performance composite scores was compared using the same penalized regression model as described above. Each participant underwent neuropsychological testing that was summarized into four composite scores summarizing the domains of episodic, semantic, working memory, and visuospatial processing and an additional overall (global) measure. Of these, episodic (*Z* = 2.42, *P* = 0.0078), semantic (*Z* = 4.85, *P* < 0.0001), visuospatial (*Z* = 5.17, *P* < 0.0001), and the global score (*Z* = 3.97, *P* < 0.0001) were significantly predicted by penalized regression after correction for multiple comparisons, whereas the working memory composite was not significantly fit by the model. The combined PET tau and Aβ topographies that best predicted each cognitive composite are shown in Fig. 4B (exact values are shown in table S3). In each case, PET tau was the dominant topography with contributions to the model, quantified as the sum of the absolute value of the regression β, two- to ninefold higher than the contribution of PET Aβ. With the exception of predicting episodic memory, the topographies were sparse, including only temporal regions and basal frontal regions. The contribution of PET tau, specifically temporal lobe tau, in each tested domain demonstrated that tau was more closely related to cognition than Aβ.

We further investigated the correlation between PET tau and Aβ topography and neuropsychological composite scores in the CDR0 group only. In the domains that were significantly associated with PET tau and Aβ topographies in the full group, we refitted the models in the CDR0 group only and calculated the correlation between the resulting predictive topographies. The topography that was predictive of episodic memory scores in the full group was not correlated with the predictive topography in the CDR0 group (*r* = 0.04, *P* = 0.70). Similarly, the topographies predictive of semantic memory performance were not significantly correlated (*r* = −0.01, *r* = 0.90). However, the topographies predicting visuospatial performance (*r* = 0.54, *P* < 0.001) and global cognitive performance (*r* = 0.19, *r* = 0.091) were correlated (although the latter showed a trend rather than significance). These data suggest that the relationship between pathology and neuropsychological performance varies over the course of the disease.

## Discussion

The present results identified topographies of tau deposition as measured by T807 PET imaging that were associated with clinical status, Aβ deposition as revealed by PET imaging, CSF AD biomarkers, and AD-related cognitive impairment. A strong correlation existed between Aβ deposited in one topography and tau deposited in another topography. Tau imaging, both globally and in a specific topography, was significantly associated with CSF measures of tau-related neural injury. In a combined model, PET tau deposition was demonstrated to be more closely associated with cognitive function than Aβ PET imaging. Together, these results establish tau imaging as an important marker of AD-related pathology.

The Aβ imaging literature has grown over the past decade (4), whereas tau imaging is a relatively new addition to the field. FDDNP [2-(1-{6-[(2-[flourine-18]flouroethyl)(methyl)amino]-2-naphthyl)-ethylidine)maltononitrile] was the first tau imaging agent but had low signal-to-noise properties and also bound to Aβ, which precluded investigation of specific pathological species (28) or required coimaging with Aβ agents and elaborate subtraction procedures (29). Methodological studies (that is, biochemistry and small-animal studies) have identified several compounds that are specific to tau pathology and have favorable biochemical properties (30, 31). Human studies, to date, have been small (*n* < 25) and have identified PET tau topographies consistent with studies in postmortem brain tissue (20, 32–35). In this preliminary body of work, PET tau pathology burden in specific ROIs (for example, hippocampus) was correlated with cognitive performance and hippocampal volume (34, 35). These studies have demonstrated the utility of tau imaging agents in identifying tau pathology burden in vivo. Our study builds on those studies by examining a larger cohort at various disease stages and capitalizing upon the spatial information of PET by explicitly relating tau topographies to disease stage, Aβ imaging, CSF biomarkers, and cognition.

PET tau and Aβ imaging acquires data at a resolution of about 5 to 8 mm and samples thousands of voxels, but the true dimensionality of the data is likely much lower (36). This study identified two topographies for both PET tau and Aβ that explained most of the variance in the data. The two tau topographies were associated with disease severity as measured by the CDR. The first tau topography was homogeneously distributed across the brain, but the second was concentrated in the temporal lobe. Similarly, the first Aβ topography was broadly distributed across the brain, whereas the second Aβ topography localized primarily in the frontal and parietal regions and was significantly associated with disease stage. Notably, the separation between CDR groups was visually more marked with respect to tau burden (Fig. 2, B and C), although significant overlap remained. Overlap with respect to CDR status likely represents the variable relationship between pathological burden and cognitive symptoms often attributed to differences in host factors, for example, cognitive reserve (8, 23). This suggests that representation of pathologically associated topographies of both tau and Aβ indexes disease progression, but the relationship to cognitive impairment is more pronounced with respect to tau.

A key feature of AD is that it is a dual proteinopathy. One of the more debated mechanisms is how two pathological species progress in independent topographies (11, 37, 38). Although the topographies are distinct, this study uses canonical correlation to emphasize that these two pathological processes are related. The correlation between tau and Aβ can be remarkably high when the relevant topographies are considered. This suggests that for a given severity of Aβ deposition, the severity of tau pathology can also be estimated. The mechanism underlying the relationship between Aβ and tau in the generation of AD is unclear, but the empirical relationship is clearly evident.

In the absence of widely used tau PET ligands, CSF-based measurements of tau pathology burden have predominated in the AD field (39). CSF-based measurements afford certain advantages (for example, broad applicability) but do not provide topographic information afforded by PET imaging. Nevertheless, CSF-based measures and PET-based measures index similar pathological processes in the context of AD. As a result, a high correlation between mean PET tau SUVR and CSF tau was expected. Perhaps less intuitive, but commonly observed, is the strong relationship between CSF tau and PET Aβ SUVR, even in the absence of neurofibrillary tangles (40). This relationship could be understood in terms of the ordered nature of biomarker progression: once detectable tau pathology is present, there is already advanced Aβ pathology, allowing for a significant relationship (8, 9). The relationship between CSF- and PET-derived measures was topographically specific. The specificity of this relationship strengthens the argument that the identified relationships are disease-specific: elevated tau in the CSF is associated with tau deposition within regions known to be sensitive to AD-related tauopathy (2). Together, these lines of evidence suggest that the same pathological process is being measured by CSF and imaging techniques.

Understanding the contribution of tau and Aβ to cognitive symptoms, particularly early in AD, has been restricted owing to the lack of topographic tau information in vivo. Here, the predictive power of PET tau and Aβ topographies on neuropsychological performance was evaluated. In each investigated cognitive domain, the weight assigned to PET tau in the best-fitting model was larger than the weight assigned to PET Aβ. This implied that tau burden, not Aβ burden, is more closely linked to cognition. This increased correlation suggests that tau and cognition are more closely linked, consistent with extant models of AD (8, 9). Furthermore, PET imaging data coupled with the present penalized regression approach also provide regional specificity. Temporal lobe tau tended to be the strongest predictor of cognitive performance consistent with the known focus of tau pathology. The selective nature of our model suggests that tauopathy severity in the temporal lobe is sufficient to predict cognitive impairment across the early disease stages investigated here.

A number of design and analytical decisions influence the interpretation of our data and resulting conclusions. First, the recent implementation of this tau tracer limits the number of participants who have been scanned. Thus, this study includes a small number of participants compared to Aβ imaging studies. However, the participants in this study were well characterized, which improves the reliability of our results. Also related to the recent development of tau tracers, the present results are cross-sectional in nature and do not capture the longitudinal course of the disease. Finally, a number of analytical decisions may influence these results. For example, tau and Aβ deposition was measured within ROIs defined by anatomical parcellation; there are other equally valid methods to define brain ROIs. The effect of these analytical decisions is unknown, but the present approach makes use of techniques previously implemented and well validated in the Aβ imaging literature (21).

Together, these data provide important insight into the relationship between tau topographies and other measures of AD pathology and cognitive symptoms. This inference is made possible by the emergence of PET tau imaging agents. Tau imaging accurately discriminates disease stage assessed by the CDR, strongly correlates with CSF measures, in particular within temporal regions, and is more closely related to cognitive performance than is Aβ imaging. Thus, tau pathology appears to be more closely linked to cognitive dysfunction consistent with existing hypotheses. These data suggest distinct roles of PET Aβ and tau imaging going forward. PET Aβ will likely remain a powerful tool for early detection of AD pathology during the preclinical period. However, our data suggest that the close relationship between PET tau and disease stage and symptomatology will be critical for tracking the efficacy of disease-modifying therapies.

## Materials and Methods

### Study design

Forty-six participants were recruited from ongoing studies of memory and aging at the Knight Alzheimer's Disease Research Center at Washington University in St. Louis. Participants underwent cognitive assessment with the CDR scale (19), and MRI and PET imaging with T807 and florbetapir. All participants with CDR>0 (that is, clinically impaired) carried clinical diagnoses of AD or mild cognitive impairment due to AD. A subset of participants also underwent lumbar puncture (*n* = 36) and neuropsychological assessment (*n* = 40). All participants or their designees provided written informed consent before participating in this study that was consistent with the regulations of the Washington University in St. Louis School of Medicine Institutional Review Board.

### Neuropsychological assessment

A comprehensive neuropsychological battery was administered to all participants, typically within 2 weeks of the annual clinical assessment. Standardized test scores were averaged to form four composites [see (41) for details on construction of composites and the standardization sample]. The episodic memory composite included the sum of the three free recall trials from the Selective Reminding Test (42), Associate Learning from the Wechsler Memory Scale (WMS) (43), and immediate recall of the WMS Logical Memory or WMS-Revised Logical Memory (44). The semantic composite included the Information subtest from the Wechsler Adult Intelligence Scale (WAIS) (45), the Boston Naming Test (46), and the Animal Naming Test (46). The working memory composite included WMS Mental Control, Digit Span Forward and Digit Span Backward, and Letter Fluency for S and P. The visuospatial composite included the WAIS Block Design and Digit Symbol subtests and Trailmaking Test A and B (47).

### Imaging

MRI and florbetapir data were acquired on a Biograph mMR scanner (Siemens Medical Solutions) in a single session. T807 data were acquired in a separate session on a Biograph 40 PET/CT (computed tomography) scanner. Participants received a single intravenous bolus injection of 10 mCi of florbetapir and a single intravenous bolus injection of 6.5 to 10 mCi of T807.

PET data were analyzed using our standard technique (18, 19) implemented in the PET unified pipeline (http://github.com/ysu001/PUP). FreeSurfer segmentation (44) (http://freesurfer.net/) was used as the basis of the quantitative analysis to obtain regional SUVR with cerebellar gray matter as the reference region. Partial volume correction was also performed using a regional spread function technique (19). For florbetapir PET, the data between 50 and 70 min after injection were used for the analysis, and for T807, the imaging data between 80 and 100 min were used instead.

### CSF analysis

CSF (20 to 30 ml) was collected after overnight fasting as described previously (48, 49). Total and phosphorylated tau and Aβ_42_ were measured using enzyme-linked immunosorbent assay [INNOTEST, Fujirebio (formerly Innogenetics)].

### Dimensionality reduction and analysis of a single PET modality

PET SUVR, for both T807 and florbetapir, can be measured at the resolution of voxels, structurally or functionally defined sets of voxels, or the entire brain. This gives rise to data sets that appear to be high-dimensional (that is, many samples per imaging session) but can be represented by lower-dimensional projections. These bases can be identified using SVD or related procedures (for example, principal component analysis). In PET data, these composite factors represent topographies (that is, the weighted combination of brain regions), and the contribution of a given topography to an individual participant's data can be estimated. SVD was performed on the regional SUVR values (as variables) across participants (as samples) for tau and Aβ separately. The number of significant factors was determined by a permutation test.

### Identifying the relationship between tau and Aβ topographies and CSF and neuropsychological performance using penalized regression

Penalized regression was used to identify the relationship between CSF tau and Aβ_42_ or neuropsychological *Z* scores (as outcome variables) and PET tau and Aβ topographies (as predictors). The penalty in penalized regression is enforced against the estimated regression βs (26). The family of penalized regression fit here is the elastic net penalty (27): the L1 penalty component enforces sparsity (that is, many βs will be identically 0, not included in the model) (26), whereas the L2 penalty allows highly correlated variables to enter the model simultaneously (27). Elastic net models are well suited for data that are highly collinear such as the regional PET data. Model parameters (total penalty and relative contribution of L1 and L2 penalty) are chosen by cross-validation. Penalized regression models, like any machine learning algorithm, are prone to overfitting. To assess significance of the fit models, permutation resampling determined the distribution of *R*^2^ values under the null hypothesis.

### Canonical correlation identifies relationships between tau and Aβ topographies

Description of the correlation between tau and Aβ topographies is accomplished using canonical correlation (50, 51). Canonical correlation is the multivariate generalization of the more familiar, for example, Pearson bivariate correlation. Canonical correlation identifies the weighted average of ROIs in one distribution that is maximally correlated with another weighted average of ROIs in another distribution. The weighting vectors, or canonical vectors, define topographies.

## Supplementary Material

Supplemental_data

Supplemental_text Fig. S1. PET tau and Aβ SUVR images from representative subjects in both the CDR0 and CDR>0 group.
Table S1. Analysis of variance (ANOVA) results related to SVD topographies.
Table S2. Mean PET and CSF correlation matrix.
Table S3. Regional regression β values.
Table S4. Comparison of PET tau SVD results.
Table S5. Leave-one-out analysis results.

## Figures and Tables

**Table 1:** Cohort demographics Demographics for the main cohort and two subsets. CSF, CSF assay; NP, neuropsychological testing; APOE, apolipoprotein ε.

	T807 + florbetapir	T807 + florbetapir + CSF	T807 + florbetapir + NP
*N*	46	36	40
Age (SD)	75.4 (6.6)	76.3 (6.6)	75.7 (6.5)
Male/female	30/16	25/11	27/13
CDR0/0.5/≥1	36/7/3	31/2/3	34/4/2
APOE ε4+	20	16	18
