# Supplementary material for: Tau and Aβ imaging, CSF measures, and cognition in Alzheimer's disease
Source: Sci Transl Med. Author manuscript; Available in PMC 2017 May 11. (PMC5267531; doi:10.1126/scitranslmed.aaf2362)
Supplement: Supplemental_text — Fig. S1. PET tau and Aβ SUVR images from representative subjects in both the CDR0 and CDR>0 group. Table S1. Analysis of variance (ANOVA) results related to SVD topographies. Table S2. Mean PET and CSF correlation matrix. Table S3. Regional regression β values. Table S4. Comparison of PET tau SVD results. Table S5. Leave-one-out analysis results. [file NIHMS830812-supplement-Supplemental_text.pdf]

Science Translational Medicine
AAAS

www.sciencetranslationalmedicine.org/cgi/content/full/8/338/338ra66/DC1

Supplementary Materials for

Tau and Aβ imaging, CSF measures, and cognition in Alzheimer's disease

Matthew R. Brier, Brian Gordon, Karl Friedrichsen, John McCarthy, Ari Stern, Jon Christensen, Christopher Owen, Patricia Aldea, Yi Su, Jason Hassenstab, Nigel J. Cairns, David M. Holtzman, Anne M. Fagan, John C. Morris, Tammie L. S. Benzinger, Beau M. Ances*

*Corresponding author. Email: bances@wustl.edu

Published 11 May 2016, *Sci. Transl. Med.* **8**, 338ra66 (2016)
DOI: 10.1126/scitranslmed.aaf2362

This PDF file includes:

Methods
Fig. S1. PET tau and Aβ SUVR images from representative subjects in both the CDR0 and CDR>0 group.
Table S1. Analysis of variance (ANOVA) results related to SVD topographies.
Table S2. Mean PET and CSF correlation matrix.
Legend for table S3
Table S4. Comparison of PET tau SVD results.
Table S5. Leave-one-out analysis results.

Other Supplementary Material for this manuscript includes the following:
(available at www.sciencetranslationalmedicine.org/cgi/content/full/8/338/338ra66/DC1)

Table S3 (Microsoft Excel format). Regional regression β values.

Supplemental Information.

Detailed Methods

Dimensionality Reduction and Analysis of a Single PET Modality

Algebraically, let $X = \mathbb{R}^{N \times M}$ be a matrix of regional SUVR values where N indexes participants and M indexes brain regions. The SVD is defined as $X = USV^T$. S is a diagonal matrix whose values are the singular values and determine the number of significant topographies. The matrix V is an $M \times M$ matrix whose columns index each topography. The matrix U is an $N \times N$ matrix whose columns index the representation of each topography in each participant. The number of significant topographies is determined by randomly shuffling X and determining a null distribution of S_{null}. Values of S that exceed the 95% confidence interval of S_{null} are determined to be significant.

Canonical Correlation Identifies Relationships Between tau and Aβ Topographies

Let $X_1, X_2 \in \mathbb{R}^{N \times M}$ be the matrices containing the tau or Aβ SUVR values where N indexes participants and M indexes ROIs. Define the covariance matrix as:

$$\Sigma = \begin{bmatrix} \Sigma_1 & \Sigma_{12} \\ \Sigma_{21} & \Sigma_2 \end{bmatrix} = \begin{bmatrix} X_1^T X_1 & X_1^T X_2 \\ X_2^T X_1 & X_2^T X_2 \end{bmatrix}$$

Due to the number of ROIs ($2M$) not being small compared to the number of participants, Σ is rank deficient. To allow for matrix inversion, a covariance regularization approach is adopted where a regularized covariance matrix is calculated as

$$\hat{\Sigma} = (1 - \omega)\Sigma + \omega I$$

where ω is the regularization parameter determined in closed form and I is the identity matrix (*52, 53*).

Let u and v be the canonical variables corresponding to X_1 and X_2, respectively. The vectors u and v can be calculated as the eigenvectors of $\hat{\Sigma}_1^{-1}\hat{\Sigma}_{12}\hat{\Sigma}_2^{-1}\hat{\Sigma}_{21}$ and $\hat{\Sigma}_2^{-1}\hat{\Sigma}_{21}\hat{\Sigma}_1^{-1}\hat{\Sigma}_{12}$ corresponding the n largest eigenvalues, respectively. The number of significant canonical correlations (i.e., n) corresponds to the rank of $\hat{\Sigma}_{12}$, which is determined by an information criterion. The i-th canonical correlation satisfies the criterion:

$$\underset{u,v}{\operatorname{argmax}}\, corr(u_i^T X_1, v_i^T X_2)$$

$$u_i \perp u_1, \cdots, u_{i-1}$$

$$v \perp v_1, \cdots, v_{i-1}$$

u_i and v_i have length M and represent topographies.

<u>Identifying the Relationship Between Tau and Aβ Topographies and CSF and Neuropsychological Performance Using Penalized Regression</u>

Penalized regression attempts to identify a relationship between an outcome of interest (i.e., CSF assay of Aβ or tau, or neuropsychological performance) and a set of predictors (i.e., regionally measured tau or Aβ). Penalized regression is used for two reasons: 1) ordinary least squares regression fails owing to the relatively large number of variables ($2M = 84$) relative to the number of participants ($N \approx 46$) and 2) penalization allows for enforcement of favorable properties on regression coefficients (e.g., sparsity of parameters). The elastic net regression is the form of

penalized regression used here. Elastic net is the linear combination of the L_1 penalized LASSO model and the L_2 penalized RIDGE regression and combines the sparsity of the former and the accommodation of highly collinear data (e.g., regional PET data) of the latter. Mathematically, elastic net regression is expressed:

$$\underset{\beta}{\operatorname{argmin}} \|\boldsymbol{y} - \beta X\|^2 + \lambda\big((1-\alpha)\|\beta\|_1 + \alpha\|\beta\|^2\big)$$

$\boldsymbol{y}$ is the outcome of interest and can either be results of the CSF assay or neuropsychological assessment. X is an $N \times 2M$ matrix where rows index participants and columns index regional tau and Aβ values. $\boldsymbol{y}$ and columns of X are made zero mean and unit variance so that the magnitudes of β are comparable. The two tunable parameters (α and λ) are determined by minimizing leave-one-out cross-validation error. $\alpha = 0$ corresponds to RIDGE regression and $\alpha = 1$ corresponds to LASSO regression. Model significance is determined by bootstrapping 1,000 null permutations of the data and comparing the error of the null models to the actual model.

Supplementary Results

PET tau SVD in expanded cohort produces similar results

To demonstrate the stability of the results presented in main text Figure 2, we repeated the tau SVD in an expanded subset of subjects who had PET tau imaging data available but not PET Aβ data. This adds two additional subjects with a CDR 1. To test whether the expand data revealed similar results we calculated the correlation between the PET tau topographies in Figure 2 with the topographies resulting from the expanded subset. The first topography was highly correlated (r =

0.99, $p < 0.001$) as was the second topography ($r = 0.99$, $p < 0.001$). Next we determined if the group differences were qualitatively similar by subjecting the expanded data to the same ANOVAs as used in the main text and Supplemental Table 1. The results were qualitatively similar in the larger cohort compared to the smaller cohort (Supplemental Table 4). These results demonstrate that these results are stable to the addition of new subjects.

PET Tau and PET Aβ SVD results are stable across subjects

To further assess the stability of the present results, we adopted a leave-one-out (LOO) approach. The test statistic is the correlation between the SVD-derived topographies in the full cohort (N=46) and the cohort less an individual. This is repeated such that every subject is left out once. If a single subject drives the results, the removal of that subject will create a low r value (i.e., the LOO result will substantially diverge from the group result). If the r values are universally high, that suggests a stable result. The results of this analysis are presented in Supplemental Table 5. The high average r value for each topography and the high minimum r value are consistent with stable results. Further, these results suggest that no single subject "drives" the present results.

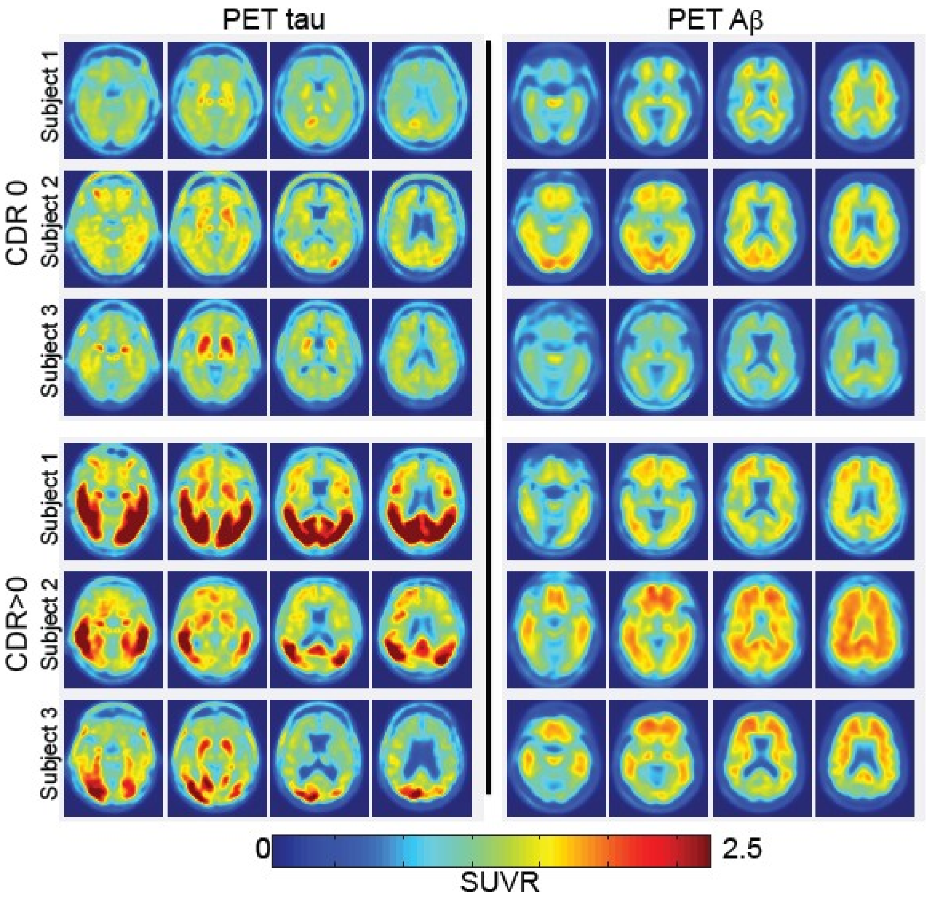

Figure S1: PET tau and Aβ SUVR images from representative subjects in both the CDR 0 and CDR>0 group.

Table S1. Analysis of variance (ANOVA) results related to SVD topographies.

	Age F	Age p	CDR F	CDR p	APOE F	APOE p
Tau 1	0.52	0.47	23.28	<0.0001	0.41	0.52
Tau 2	0.91	0.35	31.18	<0.0001	0.51	0.48
Aβ 1	2.14	0.15	1.45	0.23	0.01	0.94
Aβ 2	3.33	0.08	4.62	0.04	0.42	0.52

ANOVA table representing the effect of age, CDR status, and APOE status on the representation of the two tau and Aβ topographies described in Figure 2.

Table S2. Mean PET and CSF correlation matrix.

	Mean T807 Pet	Mean PiB Pet	CSF tau	CSF p-tau	CSF $A\beta_{42}$
Mean tau PET	1	0.45**	0.36*	0.29	–0.25
Mean Aβ PET		1	0.52**	0.49*	–0.54**
CSF tau			1	0.96**	–0.20
CSF p-tau				1	–0.13
CSF $A\beta_{42}$					1

Correlation between mean PET variables and CSF measures. *p<0.05, **p<0.01.

Table S3: Regional regression β Values.
XLSX Attached

Table S4. Comparison of PET tau SVD results.

		Age F	Age p	CDR F	CDR p	APOE F	APOE p
N=46	0.52	0.47	23.28	<0.0001	0.41	0.52	0.52
	0.91	0.35	31.18	<0.0001	0.51	0.48	0.91
N=48	Tau 1	0.01	0.94	14.32	0.0005	0.6	0.44
	Tau 2	0.06	0.80	16.91	0.0002	0.18	0.67

ANOVA table representing the effect of age, CDR status, and APOE status on the representation of the two tau topographies in the two cohorts. The N=46 cohort is reproduced from Supplemental Table 1. The N=48 cohort is the N=46 cohort with two additional CDR1 subjects.

Table S5. Leave-one-out analysis results.

	Mean r value	Min r value
Tau 1	0.99	0.99
Tau 2	0.99	0.89
Aβ 1	0.99	0.97
Aβ 2	0.98	0.96
